# When, which and how to switch: Navigating JAK inhibitors in myelofibrosis

**DOI:** 10.1111/bjh.19929

**Published:** 2024-11-27

**Authors:** Jennifer O'Sullivan, Imran Omerdeen, Bethan Psaila

**Affiliations:** ^1^ MRC Weatherall Institute of Molecular Medicine University of Oxford Oxford UK; ^2^ Department of Haematology Guy's and St Thomas' NHS Foundation Trust London UK; ^3^ New College Oxford and Oxford University Medical School Oxford UK; ^4^ Department of Haematology Oxford University NHS Trust Oxford UK; ^5^ Oxford Ludwig Cancer Institute Oxford UK

**Keywords:** clinical haematology, megakaryocytes, myelofibrosis, myeloproliferative disorder, therapy

## Abstract

Navigating choice of JAK inhibitor (JAKi) therapy for patients with myelofibrosis who are JAKi‐naïve and for those who have previously been treated with a JAKi.

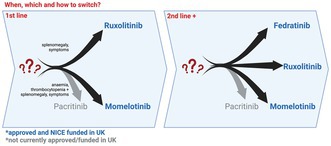

Following its approval in 2011, ruxolitinib, a Janus Kinase (JAK) 1 & 2 inhibitor, rapidly became standard of care, offering major clinical benefits for patients with myelofibrosis. Other JAK inhibitors (JAKis) have since been developed, expanding the treatment armamentarium. As of 2024, three JAKis are approved in the United Kingdom—ruxolitinib, momelotinib and fedratinib. This Nutshell review outlines our approach to navigating *when* to treat, *which* JAKi to choose as first‐line therapy, *how to switch* and future perspectives including combination therapies (Figure 1).

1. *Ruxolitinib and momelotinib* are JAK1/JAK2i approved as first‐line therapies. They reduce splenomegaly, symptoms and may confer a survival benefit, although they are not selective for the cancer clone.
2. *Momelotinib* also inhibits activin A receptor type 1 (ACVR1) thereby alleviating inflammation‐induced anaemia and is licenced in severe thrombocytopenia (platelets ≥25 × 10^9^/L).
3. *Fedratinib* is a JAK2‐selective inhibitor licenced in the United States and recently approved for funding by the National Institute of Clinical Excellence (NICE) in the United Kingdom for use as a second‐line JAKi.
4. Patients can be safely switched directly from ruxolitinib to momelotinib without transition, but transition from either ruxolitinib or momelotinib to fedratinib requires careful management due to the withdrawal of JAK1 inhibition.
5. Future regimens are likely to involve a combination of JAKi with additional agents to achieve better disease modification, and/or novel JAKi that are selective for oncogenic versus wild‐type MPL‐JAK‐STAT signalling.

## WHEN TO TREAT WITH A JAKi, AND WHICH AGENT?

*Case 1*: A 75‐year‐old man with drenching sweats, weight loss and painful splenomegaly measuring 14 cm below the costal margin. Investigations confirmed haemoglobin (Hb) 116 g/L, platelets 195 × 10^9^/L and primary myelofibrosis with *JAK2*V617F and *ASXL1* mutations.

*Case 2*: A 73‐year‐old woman with a history of calreticulin (CALR, Type I 52 bp deletion)‐driven essential thrombocythaemia (ET) develops marked fatigue and splenomegaly (8 cm below the costal margin). Investigations show Hb 83 g/L, platelets 95 × 10^9^/L and progression to post‐ET myelofibrosis.

*Case 3*: A 64‐year‐old asymptomatic man is found to be anaemic with Hb 108 g/L and splenomegaly (6 cm below the costal margin) on an annual health check and diagnosed with mutCALR‐driven primary myelofibrosis (Type II 5 bp insertion mutation).

Ruxolitinib was approved only 6 years after the discovery of *JAK2*V617F as the most common genetic lesion in myeloproliferative neoplasms (MPNs). Its approval was based on efficacy and safety data accrued in the Phase 3 COMFORT‐I and COMFORT‐II trials.^1^, ^2^ Together, these studies involved >500 patients, generating pivotal data that established the efficacy of JAKi in reducing splenomegaly and symptoms in patients with myelofibrosis. Between 30% and 40% patients benefitted from a ≥35% reduction in spleen volume, and around half experienced a ≥50% improvement in symptoms and quality of life. Subsequent pooled analyses of 3‐ and 5‐year follow‐up data suggested that these benefits translated to improved survival,^3^ with a median survival of 5.3 years in ruxolitinib‐treated patients versus 3.8 years in non‐JAKi‐treated controls (*p* = 0.0065).^4^ A similar survival benefit was observed in a real‐world study of >1000 patients.^5^ However, the original studies were insufficiently powered to estimate survival outcomes, and the survival benefit of JAKi remains contentious.^6^, ^7^

In 2024, momelotinib, a second‐generation JAKi, was approved in the United Kingdom for both first‐ and second‐line treatment of patients with symptomatic myelofibrosis and anaemia. In the setting of first‐line therapy, the SIMPLIFY‐I study investigated a head‐to‐head comparison of momelotinib and ruxolitinib in JAKi‐naïve patients. This study observed that momelotinib was non‐inferior to ruxolitinib in terms of spleen response, but a higher proportion of patients achieved a 50% reduction in symptoms with ruxolitinib.^8^ In addition to JAK1 and JAK2, momelotinib also inhibits ACVR1, a key regulator of iron homeostasis. Inhibition of ACVR1 reduces hepcidin, releasing iron stores, thereby ameliorating inflammation‐related anaemia, with approximately one in three patients achieving an anaemia benefit. Significantly more patients receiving momelotinib in SIMPLIFY‐I were transfusion independent at week 24 (66.5%) compared with patients receiving ruxolitinib (49.3%, *p* < 0.001),^8^ This study also showed a 5‐year survival probability of 55% for momelotinib‐treated patients, and no differences in survival between patients receiving momelotinib versus ruxolitinib as their first‐line JAKi.^9^ Momelotinib, therefore, presents a new option for alleviating splenomegaly, symptoms and anaemia in myelofibrosis and is an effective alternative to traditional approaches for myelofibrosis‐related anaemia such as steroids and danazol.^10^

Although both ruxolitinib and momelotinib can cause thrombocytopenia, this is more common with ruxolitinib. Safety and efficacy of momelotinib in patients with marked thrombocytopenia has been demonstrated,^11^ and momelotinib is licenced for use in anaemic patients with platelets ≥25 × 10^9^/L, whereas ruxolitinib is licenced for use with a platelet count ≥50 × 10^9^/L. Thrombocytopenia adverse events were reported in 18.7% vs. 29.2% of momelotinib‐ and ruxolitinib‐treated patients in SIMPLIFY‐1 respectively.^8^ Thrombocytopenia can limit clinically effective ruxolitinib dosing, as >10 mg twice daily is usually required for meaningful spleen and symptom responses and increased dose intensity correlates with better response. Pacritinib, a JAK2/IRAK1/FLT3/AVCR1 inhibitor, also has a particular indication for patients with thrombocytopenia and is approved in the United States for patients with platelet counts <50 × 10^9^/L but only available in the United Kingdom at present via the PACIFICA clinical trial (NCT03165734). Fedratinib has also shown safety and efficacy in those with thrombocytopenia (platelets 50–100 × 10^9^/L).^12^

### When to treat and which agent first line

These trials robustly confirmed the clinical benefits of ruxolitinib and momelotinib as first‐line JAKi. Current trial data suggest superiority of ruxolitinib for symptom control and tolerability, while momelotinib has the added benefits of improving anaemia, potentially alleviating transfusion dependence, and a lower incidence of thrombocytopenia. Therefore, a patient such as Case 1 who presents with profound symptoms and robust counts may be more suited to ruxolitinib first line, while patients in whom cytopenias predominate (Case 2) may be more appropriate for first‐line momelotinib.

### Asymptomatic patients

There is no clear evidence that JAKi therapy is beneficial in patients who are asymptomatic without problematic splenomegaly, as in Case 3. Regular monitoring is recommended, including quantitative symptom assessment and spleen measurements, and, if appropriate, control of myeloproliferation, for example, with pegylated interferon alpha, which may also reduce the mutant allele burden especially for JAK2V617F+ MPNs, and/or consideration for clinical trials.

## WHEN TO SWITCH TO A SECOND‐LINE JAKi, AND WHICH JAKi TO CHOOSE IN THE SECOND‐LINE SETTING?

*Case 1*: This patient started ruxolitinib 15 mg BD and had an excellent initial response with resolution of sweats, improved appetite and a reduction in splenomegaly from 14 to 3 cm. After 8 weeks of therapy, he became anaemic (Hb 78 g/L), requiring a red cell transfusion.

### Anaemia in the first 3 months after ruxolitinib initiation

Ruxolitinib frequently causes anaemia in the first few months after treatment initiation, although Hb levels often return to baseline after a 12‐week nadir. Haemoglobin levels can be supported with erythropoietin therapy, temporary dose reduction or red cell transfusions and anaemia in the first few months does not necessarily require a change of JAKi therapy. However, if problematic cytopenias are prolonged or emerged later during therapy, switching to momelotinib may be beneficial.

*Case 1*: His initial anaemia improved with EPO and two red cell transfusions, and he continued on ruxolitinib enjoying an excellent quality of life for 2 years returning to cycling holidays and caring for his grandchildren. However, 2.5 years after treatment initiation, his sweats returned, with no improvement following dose increase to 25 mg BD. He began to lose weight, and his spleen enlarged again to 13 cm below the costal margin.

### Options for second‐line therapy

The majority of patients discontinue ruxolitinib after 3–5 years due to loss of response, disease progression or late cytopenias. Patients discontinuing ruxolitinib have a poor survival (median survival ~1 year).^13^ Fedratinib is equally potent to ruxolitinib as a JAK2 inhibitor and, in addition, inhibits FLT3 and bromodomain and extra‐terminal motif [BET] protein BRD4, but not JAK1. The JAKARTA and JAKARTA‐2 trials confirmed that fedratinib reduced splenomegaly and improved symptoms in both frontline treatment and after ruxolitinib failure,^14^ where around half patients achieved a spleen response. These studies led to approval of fedratinib as a first‐ and second‐line therapy in the United States, but it is currently only funded as a second‐line JAKi in the United Kingdom.

The choice of JAKi in the second‐line setting is guided by the clinical features and goals of therapy, just as in the first‐line setting. For patients in whom reducing disease‐related splenomegaly is the primary goal and this has not been achieved despite maximum tolerated doses of ruxolitinib, fedratinib may be considered. In the recently reported FREEDOM‐2 study, a significantly higher proportion of patients who switched from ruxolitinib to fedratinib achieved a 35% reduction in spleen size than those who remained on best available therapy (BAT, largely continued ruxolitinib, 36% vs. 6%, *p* < 0.001).^15^ A clinical benefit of fedratinib has also been shown in the real‐world setting.^16^ To date a randomised, controlled comparison of momelotinib versus fedratinib in ruxolitinib‐treated patients has not been performed. The SIMPLIFY‐2 study comparing momelotinib versus BAT (again largely continued ruxolitinib) in patients previously treated with ruxolitinib found that switching to momelotinib was not superior to BAT for achieving a ≥35% reduction in spleen size from baseline.^17^ A consideration for patients in the United Kingdom is that NICE funding is provided for a patient to return to ruxolitinib after switching to momelotinib regardless of how long the patient has been off ruxolitinib, if the clinical benefit of ruxolitinib was superior. However, funding is not provided for patietns return to ruxolitinib after switching to fedratinib if ruxolitinib was discontinued for more than 3 months.

Patients for whom anaemia and/or thrombocytopenia are the dominating features are more suited to momelotinib than fedratinib in the second‐line setting. Both the SIMPLIFY‐2 study^17^ and the MOMENTUM study (comparing momelotinib vs. danazol in JAKi‐exposed patients with symptomatic anaemia^18^) showed a significant anaemia benefit of momelotinib in the second‐line JAKi setting. In a subgroup analysis of anaemic patients in SIMPLIFY‐2, transfusion independence at week 24 was observed in 33% momelotinib‐treated compared with 12.8% of those receiving standard therapy (largely ruxolitinib).^19^

### How to transition between JAKi

Patients can transition directly between ruxolitinib and momelotinib without tapering or wash‐out.^20^ However, transition from either ruxolitinib or momelotinib to fedratinib requires overlap and dose tapering (+/− steroid cover), due to the cessation of JAK1 inhibition and fedratinib's long half‐life which may cause a rebound cytokine storm.

### Notable toxicities

Prior to JAKi treatment initiation, all patients should be counselled regarding the increased incidence infections including shingles and other opportunistic infections, and the increased incidence of non‐melanoma skin malignancies with JAKi therapy, which has been clearly documented for ruxolitinib but may occur with other JAKi.^10^ A non‐live shingles vaccine and/or acyclovir prophylaxis and dermatology monitoring are recommended. Momelotinib and fedratinib can cause mild/moderate gastrointestinal (GI) disturbances. These can be pronounced with fedratinib, requiring anti‐emetic/diarrhoeal medication. Monitoring of lipase and amylase is advised, as increased levels of these enzymes can occur with momelotinib^21^ and fedratinib.^22^ Momelotinib is associated with a peripheral neuropathy risk that may not be reversible.^21^ During the clinical development of fedratinib, four cases of thiamine deficiency‐related encephalopathy were reported, leading to a recommendation for monitoring of blood thiamine and thiamine supplementation during treatment.

*Case 1*: The patient weaned off ruxolitinib over 2 weeks, transitioned to fedratinib 400 mg OD with thiamine 100 mg OD and required ondansetron and loperamide for side effect control. His spleen reduced from 13 to 7 cm but had only a modest improvement in symptoms. He developed thrombocytopenia requiring dose reduction to 200 mg OD and 8 months later became transfusion‐dependent. Momelotinib was not available at this time. He died within a year of ruxolitinib cessation.

## FUTURE DIRECTIONS AND RESEARCH

We are now in an era of choice of JAKi in myelofibrosis, improving our ability to improve the quality of life and control key disease features. However, while JAKis are likely to remain the cornerstone of myelofibrosis therapy, current agents do not specifically target the malignant clone, or substantially alter the tempo of disease progression. On the horizon are new approaches aiming to achieve benefits beyond spleen and symptom control. Many of these agents are being tested as add‐ons to JAKi. The use of erythropoiesis‐stimulating agents in combination with JAKi is well established, and JAKis combined with interferon have the potential to reduce *JAK2*V617F allele burden while controlling splenomegaly and symptoms.^23^, ^24^ Other novel agents in the pipeline are targeting inflammation (BET inhibitors), the p53 pathway (MDM2), apoptosis regulators (Bcl‐2/Bcl‐XL) and TGFβ signalling.^25^ Also emerging are mutant CALR‐directed immunotherapies and JAKis that are more selective for JAK2V617F over wild‐type JAK2. These advances are likely to substantially improve disease control and will hopefully transform the future treatment landscape for myelofibrosis.

**FIGURE 1:**
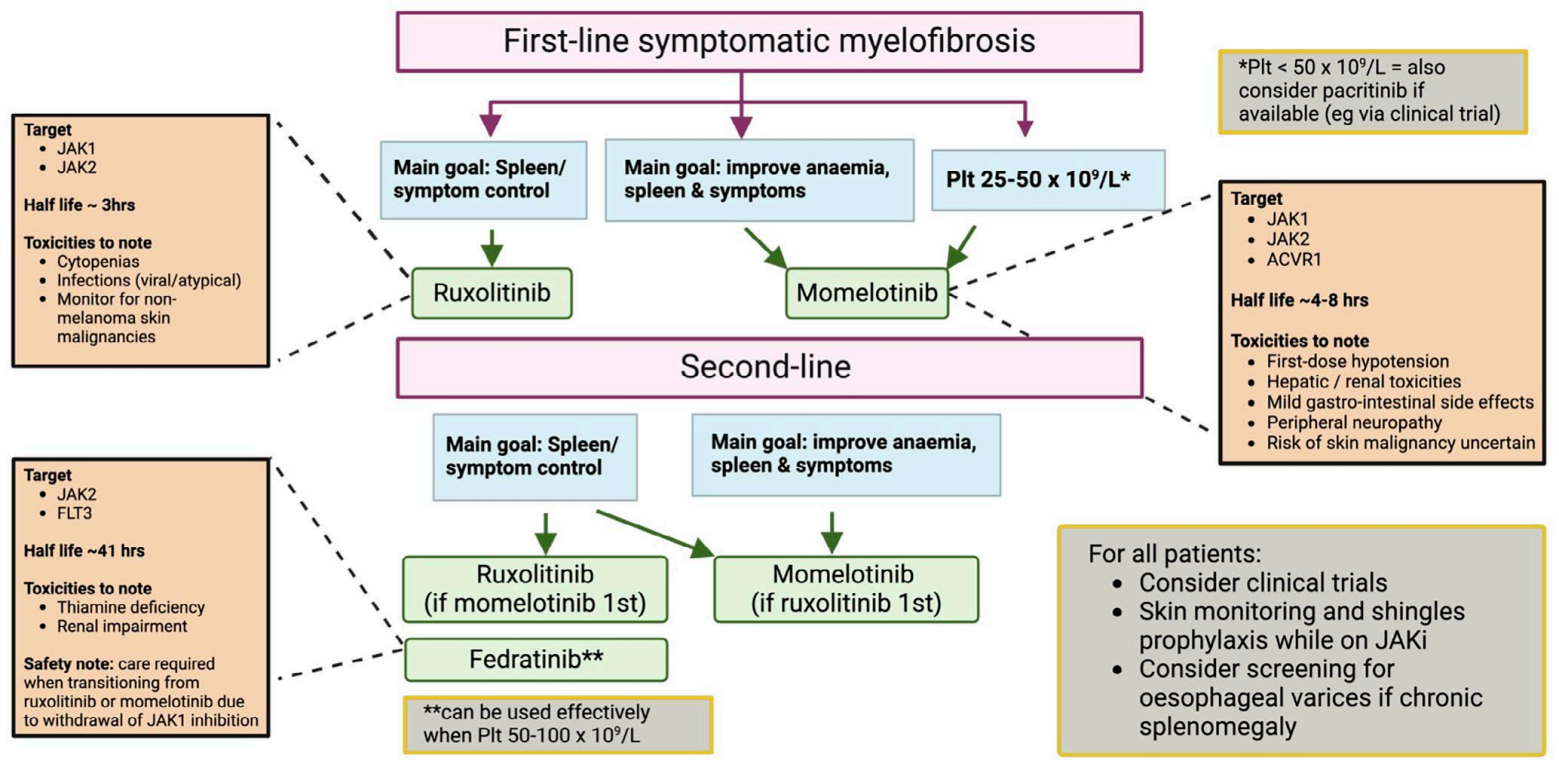
Our approach to choosing a JAK inhibitor in myelofibrosis. Plt = platelets; JAKi = JAK inhibitor.

## FUNDING INFORMATION

BP receives funding from Cancer Research UK (Senior Fellowship), the Oxford Ludwig Institute for Cancer Research (Associate Member) and the National Institute for Health Research (NIHR) Oxford Biomedical Research Centre (BRC) Cancer Theme.

## CONFLICT OF INTEREST STATEMENT

JOS has received honoraria for consulting and/or speaking engagements from Constellation Therapeutics, Novartis, Karyopharm & Medscape. BP has received research funding from Alethiomics, Incyte and Galecto Ltd, honoraria for consulting and/or speaking engagements from Alethiomics, Incyte, Blueprint Medicines, GSK, BMS and Novartis and is a co‐founder, board member and major shareholder in Alethiomics.
